# Parliament and the Profession

**Published:** 1918-06-29

**Authors:** 


### PARLIAMENT AND THE PROFESSION.

### Salvarsan Substitute.

Mr. Hayes Fisher informed Mr. Snowden that arrangements have been made for the manufacture of kharsivan, neokharsivan, arsenobillon, novarsenobillon, diarsenal, and galyl under the conditions contained in licences issued by the Board of Trade, and are tested by the Medical Research Committee before sale. The preparations are supplied by local authorities, free of charge, to registered medical practitioners who possess qualifications required by the Local Government Board.

### Army Dental Service.

Mr. Macpherson stated that the number of dental surgeons employed in the Royal Army Medical Corps is about to be largely increased. Of the 640 dentists at present holding commissions 96 are serving in France.

### Military Nurses' Uniforms.

The Financial Secretary to the War Office, Mr. Forster, informed Mr. Chancellor that nurses of the Queen Alexandra's Imperial Military Nursing Service and the Territorial Force Nursing Service serving at home are entitled to an allowance for uniform of £8 per annum. In practice the first year's grant includes an advance of £4 in respect of the second. A further allowance of £1 per annum is made when a nurse proceeds abroad.

### Tuberculosis in the Army.

Mr. Macpherson informed Mr. Chancellor that special investigation had been made in respect of men invalided from the British Army through tuberculosis, and particular attention directed to any possible influence of inoculations and vaccinations on tuberculosis. The result shows that there is no evidence whatever that inoculation or vaccination, as at present carried out in the Army, has any deleterious effect of the nature suggested.

